# Mélanome malin vulvaire: à propos d’un cas observé à l’Hôpital du Cinquantenaire de Lubumbashi

**DOI:** 10.11604/pamj.2020.36.124.19138

**Published:** 2020-06-25

**Authors:** Gabriel Kapya Mukeya, Ivan Mwandwe Kakoka, Joseph Chola Mwansa, Willy Arung Kalau

**Affiliations:** 1 Service de Gynécologie-Obstétrique, Hôpital du Cinquantenaire de Lubumbashi, Lubumbashi, République Démocratique du Congo,; 2 Département de Gynécologie-Obstétrique, Faculté de Médecine, Lubumbashi, Université de Lubumbashi, Lubumbashi, République Démocratique du Congo,; 3 Service de Chirurgie, Hôpital du Cinquantenaire de Lubumbashi, Lubumbashi, République Démocratique du Congo,; 4 Département de Chirurgie, Faculté de Médecine, Université de Lubumbashi, Lubumbashi, République Démocratique du Congo

**Keywords:** Mélanome malin vulvaire, hémivulvectomie, lymphadénomectomie superficielle, Malignant vulvar melanoma, hemivulvectomy, superficial lymphadenectomy

## Abstract

Le mélanome malin primitif de l'appareil génital féminin est une tumeur extrêmement rare. Il est néanmoins plus observé à la vulve qu’au col utérin et au vagin. La localisation vulvaire représente environ 1% (toutes localisations confondues) des mélanomes, puis par ordre de fréquence la localisation vaginale, utérine puis ovarienne; et moins de 200 cas de cancers de la vulve ont été décrits à travers le monde. Nous présentons ici un cas clinique de mélanome malin vulvaire chez une femme ménopausée de 72 ans. Une hemivulvectomie partielle et une lymphadénomectomie superficielle inguinale bilatérale ont été réalisées. Les suites postopératoires étaient globalement favorables et la patiente a quitté le service au 15^e^ jour post opératoire.

## Abstract (en)

Primary malignant melanoma of the female genital tract is an extremely rare tumor. It most commonly affects the vulva then the cervix and the vagina. Vulvar cancer accounts for about 1% (all sites) of melanomas. In order of frequency, it occurs in the vagina, uterus and ovary. Less than 200 cases of vulvar cancers have been described worldwide. We here report a clinical case of malignant vulvar melanoma in a 72-year-old postmenopausal woman. Partial hemivulvectomy and bilateral superficial inguinal lymphadenectomy were performed. The postoperative course was favorable and the patient was discharged on postoperative day 15.

## Introduction

Les cancers vulvaires sont rares et représentent 2 à 7% de tous les cancers gynécologiques. Ils apparaissent principalement après la ménopause. Les carcinomes squameux sont les plus fréquents et représentent environ 90% de tous les cancers vulvaires. Le mélanome malin vulvaire est le deuxième sous type (5 à 10%) de tous les cancers vulvaires et moins de 200 cas ont été décrits à travers le monde [1-3]. L’intérêt que l’on porte sur cette pathologie réside dans le fait qu’elle soit moins connue à cause de sa rareté et affecte les patientes ménopausées qui souvent ne sont pas prêtes à déclarer dans un délai acceptable leurs pathologies. Par ailleurs, quoi que rare, elle mérite d’être diagnostiquée par le gynécologue le plus précocement possible [4,5]. En outre, son traitement a évolué d’une chirurgie très invasive avec forte morbidité postopératoire vers une chirurgie moins invasive améliorant les suites opératoires et selon les cas accompagnés par une radiothérapie avant et ou après la chirurgie [6-8]. Nous présentons ici un cas clinique de mélanome malin vulvaire d’une ménopausée de 72 ans qui a consulté plusieurs mois après l’apparition des premiers symptômes et qui a bénéficié d’une chirurgie moins invasive qu’il y a plus de cinq ans et dont les suites postopératoires immédiates étaient satisfaisantes.

## Patient et observation

Malade K.K, âgée de 72 ans, consulte en date du 12 mars 2019 pour un prurit et une tuméfaction au niveau de la vulve gauche apparus depuis 8 mois. Comme automédication, elle a appliqué mais sans succès des bains chauds sur la lésion. Au contraire les symptômes s’aggravaient et il s’y est ajouté une incontinence urinaire. La patiente a eu sa ménarche à 13 ans et s’est mariée à l’âge de 21 ans. Elle est mariée, mère de 12 enfants. Le dernier enfant a 26 ans. Elle ne fume pas et ne prend pas d’alcool. Elle n’est ni hypertendue ni diabétique. Elle n’a jamais été hospitalisée ni opérée. Elle est ménopausée depuis 19 ans.

**Figure 1:**
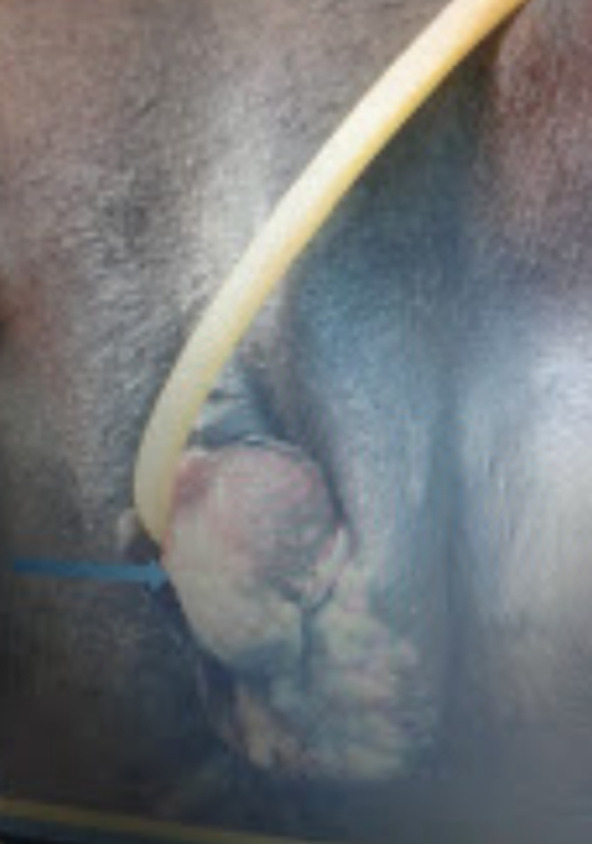
le mélanome vulvaire en préopératoire

**A l’examen clinique, nous avons constaté:** 1) **à l’examen physique général:** a) un amaigrissement (poids de 46 kg contre 60 kg en 2018, taille 1,60 m indice de masse corporelle (IMC)=14,37), b) une pression artérielle de 118/72 Hg mm de mercure, c) une fréquence cardiaque de 98 battements la minute, d) une fréquence respiratoire de 24 cycles par minutes, e) une température de 36,1°, f) les conjonctives palpébrales étaient bien colorées, les conjonctives bulbaires anictériques, g) la sphère cardio-pulmonaire était sans particularité, h) l’abdomen était souple, indolore, sans hépato-splénomegalie mais on a noté des adénomegalies inguinales bilatérales et indolores: deux adénopathies à droite et trois à gauche d’environ 1,5 à 3 cm de diamètre. 2) **A l’examen gynécologique, nous avons observé:** a) une tuméfaction vulvaire à gauche, d’environ 7x4 cm de diamètre, irrégulière, de consistance dure, indolore, intéressant la petite et la grande lèvre gauches, avec une légère induration péri-anale (Figure 1). b) A l’examen au speculum, le col était sain mais atrophique, la muqueuse vaginale était saine. C) Au toucher vaginal, on a palpé un petit utérus en antéversion et antéflexion, ferme, indolore, les annexes étaient libres et indolores. La masse infiltrait la paroi vaginale homolatérale, sur 1 cm de longueur. D) Au toucher rectal, la cloison recto vaginale était libre.

Devant ce tableau clinique, nous avons évoqué le diagnostic d’un cancer de la vulve au stade III b selon la classification de la FIGO. Une biopsie de la lésion a été pratiquée sous narcose pour examen histopathologique. Celui-ci a conclu à un mélanome malin de la vulve. La radiographie et l’échographie abdomino-pelviennes n’ont révélé aucune lésion métastatique. Mais à l’échographie de la vulve, on a noté une masse solide, aux contours irréguliers, hyper vascularisée et mesurant en profondeur 43x23mm de diamètre. Le bilan préopératoire était satisfaisant: a) Hémoglobine: 11,9g/dl, b) Glycémie à jeun: 82mg/dl, c) Temps de saignement: 2 minutes, d) Temps de coagulation: 4,2 minutes.

**Figure 2:**
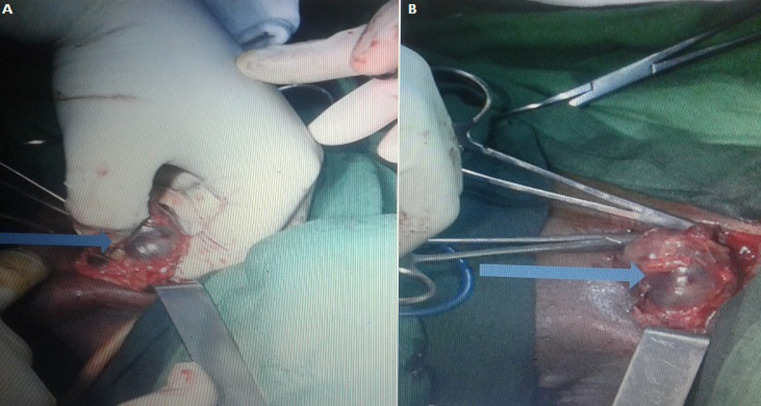
les ganglions inguinaux (A, B)

**Figure 3:**
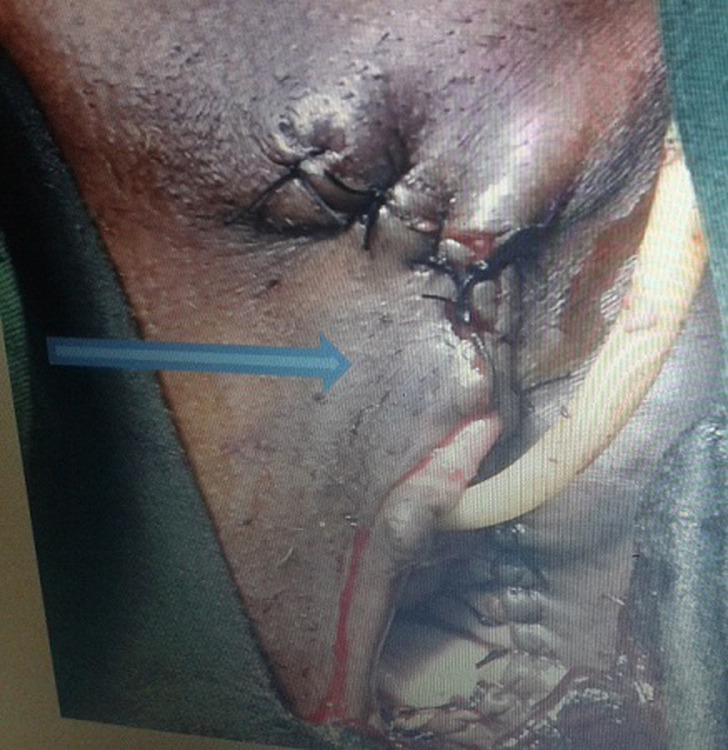
vulve en postopératoire immédiat

La sérologie VIH était négative. Les fonctions rénales et hépatiques étaient normales; l’électrocardiogramme était normal. L’intervention chirurgicale a lieu le 20 mars 2019 sous rachianesthésie. Elle a consisté à travers 3 incisions en une hémi- vulvectomie gauche élargie à la paroi vaginale homolatérale (l’incision passant à 2 centimètres autour de la lésion) et en une lymphadénomectomie inguinale bilatérale (2 ganglions à droite, 3 ganglions à gauche d’environ 1.5 à 3 cm de diamètre, de coloration noirâtre) (Figure 2). L’exérèse de la masse en profondeur s’est effectuée jusqu’au rectum avec ouverture de ce dernier. La brèche anale a été réparée distinctement de la cloison recto-vaginale et du vagin. Après l’exérèse de la masse et la réparation de l’incision, la vulve s’est présentée comme cela est observé sur la Figure 3. L’examen anatomopathologique de la masse de coloration brunâtre mais de contenu noirâtre et des adénopathies inguinales a confirmé le diagnostic de mélanome vulvaire avec atteinte ganglionnaire, sans stigmates de human papillomavirus (HPV). Les suites opératoires ont été simples malgré une petite fistule recto-vaginale qui a été réparée. L’évolution a été satisfaisante et la patiente a quitté l’hôpital à la troisième semaine postopératoire. Un bilan contrôle et des séances de radiothérapie ont été planifiées au cas où cela s’avérait nécessaire.

## Discussion

Le cas que nous présentons est celui d’une patiente de 72 ans, ménopausée qui a présenté un mélanome de la vulve. Le mélanome malin primitif de l'appareil génital féminin est une tumeur extrêmement rare. Il est néanmoins plus observé à la vulve qu’au col utérin et au vagin [1]. Dans notre clinique, c’est le premier cas de mélanome que nous avons observé après quatre ans de pratique. Ce qui semble confirmer la rareté de la pathologie. Les mélanomes du tractus génital féminin représentent une localisation rare (moins de 2% des mélanomes toutes localisations confondues). Mais la localisation vulvaire est la plus fréquente comme le décrit Jane-ChloéTrone *et al*. [2]. Cette localisation vulvaire représente environ 1% des mélanomes puis par ordre de fréquence la localisation vaginale, utérine puis ovarienne. Ces cancers ont un pronostic péjoratif, si le diagnostic est tardif. La patiente que nous présentons avait 72 ans. Certains auteurs l’ont diagnostiqué à un âge supérieur à 60 ans lors du diagnostic [3]. Ceci pourrait s’expliquer par le fait que ces femmes âgées ont du gène à déclarer la pathologie et en retardent le diagnostic. Il s’écoule souvent un grand délai avant le diagnostic de la maladie. Ce fait est imputable au retard par la malade à consulter et à la méconnaissance des symptômes de la maladie dans le chef des malades et de certains cliniciens. Ce qui diminue considérablement le taux de survie [4,5].

Par ailleurs, c’est une entité survenant soit en pré ou post ménopause, ce qui correspond à l’âge de diagnostic. Avec l’augmentation de l’espérance de vie, on peut s’attendre à rencontrer plus fréquemment ce type de cancers. Devant la gravité de cette pathologie, la connaissance de ses symptômes par les malades et les cliniciens entrainera un diagnostic précoce, gage de la survie des patientes dans notre milieu à faibles ressources. Le diagnostic était fondé sur la clinique et l’examen histopathologique. Ce dernier avait révélé un épithélium hyperplasique pseudocarcinomateux. Le derme était d’une prolifération cellulaire faite des cellules arrondies au cytoplasme large par endroit et eosinophile; un infiltra inflammatoire abondant et des signes de pigmentation ont été observés au niveau cutané; ce qui suggérait un mélanome malin. Certes, Ces tumeurs sont de diagnostic histopathologique. L’étiologie des cancers vulvaires est inconnue mais certains facteurs de risques sont incriminés notamment l’âge avancé, le lichen scléreux surtout chez les personnes âgées, l’infection HPV chez les femmes plus jeunes, la maladie de Paget, le tabagisme, l’immunodépression ou une histoire de néoplasie cervicale. Des recherches poussées montrent l’implication de la génétique. En effet, le profil génétique particulier des Mélanomes vulvaires et vaginales justifie la recherche des mutations des gènes c-KIT et NRAS en plus de BRAF. La présence des mutations c-KIT pourrait justifier l’emploi d’inhibiteurs des tyrosines kinases spécifiques de c-KIT, et celle des mutations NRAS l’utilisation d’inhibiteurs de MEK [6]. Le prurit représente le symptôme le plus fréquent du cancer vulvaire suivi de la présence d’une masse vulvaire comme c’est fut le cas dans cette description clinique.

Il a été réalisé une hémivulvectomie gauche et une lymphadénomectomie bilatérale. Au Centre Hospitalo-Universitaire de Rabat, au Maroc, plus de la moitié des 83 patientes prises en charge de janvier 2007 à janvier 2008, a bénéficié d’une vulvectomie totale ou partielle et un curage ganglionnaire bilatéral (dans 32% de cas), et unilatéral (dans 28% de cas); le traitement étant essentiellement chirurgical [7]. Selon la Société Japonaise d’oncologie [8], la chirurgie est le premier choix de traitement et il y a eu au Japon, un virage du traitement de la radiothérapie vers la chirurgie comme premier choix. Cependant, les patientes étant souvent âgée et à cause du risque et des complications opératoires, la radiothérapie peut avoir la primeur. Dans les cas de risque élévé de metastases, la radiothérapie est utilisée en adjuvant. En ce qui concerne le traitement chirurgical, il y a plus de 5 ans, la tendance était de réaliser une vulvectomie totale et une lymphadenomectomie systémique allant jusqu’au pelvis, actuellement on se limite à une chirurgie localisée sur la zone malade et réaliser une lymphadénomectomie superficielle (inguinale bilatérale ou homolatérale) à cause d’une morbidité induite par la chirurgie large. C’est cette dernière technique qui a été utilisée au cours de cette intervention. Elle a l’avantage de réduire la morbidité postopératoire. En fait c’est une chirurgie avec lymphadénomectomie sélective qui consiste en l'exérèse du ou des ganglions fixant l'isotope ou colorés de la chaîne inguinale superficielle voire profonde, ainsi que les ganglions palpables ou palpés en peropératoire, sans réaliser de véritable curage inguinal complémentaire. La lymphadénomectomie sélective est donc susceptible de réduire la morbidité, en orientant les prélèvements vers les ganglions drainant la zone tumorale, sans risquer les complications liées au curage inguinal [9].

La lymphadénomectomie fait recours à la médecine nucléaire permettant de réaliser la lymphoscintigraphie pour la détection des ganglions sentinelles [10]. Par ailleurs, près de la moitié des mélanomes cutanés portent des mutations activant BRAF et peuvent donc être traités avec des inhibiteurs de BRAF kinase tels que le dabrafenib (150 mg deux fois par jour), souvent en association avec un inhibiteur de la MEK tel que le trametinib (2 mg une fois par jour). Dans un essai de phase III mené auprès de 704 patients, l'association d'un inhibiteur de BRAF et de MEK était associée à un taux de succès de 64% et à une survie sans progression médiane de 11,4 mois, par rapport à un taux de réponse de 51% et à une survie sans progression médiane de 7,3 mois pour un inhibiteur de BRAF seul. Les mélanomes muqueux présentent rarement des mutations dans BRAF et ne sont donc généralement pas une option pour ces patients. Cependant, toutes les patientes atteintes de mélanome de la muqueuse doivent subir un test de mutation du gène BRAF. Si une mutation du gène BRAF activant est détectée chez un patient atteint d'un mélanome de la muqueuse, l'association d'un inhibiteur de BRAF et de la MEK doit alors être envisagée [11]. Le mélanome malin vulvaire est particulièrement agressif et a un pronostic sombre. L’extension de la tumeur se fait principalement par voie lymphatique mais elle peut avoir lieu aussi de proche en proche et par voie sanguine. La survie globale à 5 ans varie entre 8 et 50% et diminue encore de moitié en cas d’atteinte lymphatique inguinale. Les facteurs pronostiques sont le diamètre de la tumeur, la profondeur de l’invasion, la différentiation de la tumeur, sa marge chirurgicale, l’âge avancé de la patiente, l’atteinte de l’urètre, du vagin, du périnée et surtout des ganglions inguinaux. En effet, en l’absence d’atteinte ganglionnaire la survie globale à 5 ans est de 90%, elle tombe à 50% en cas d’atteinte positive. Le nombre des ganglions positifs est d’une importance critique. S’il y a trois ganglions positifs ou plus et en cas de bilatéralité, la survie globale à deux ans n’est plus que de 20% [12,13].

## Conclusion

En somme, il s’est agi d’une patiente ménopausée de 72 ans qui a présenté un mélanome malin vulvaire chez qui une hémivulvectomie et un curage ganglionnaire inguinal superficiel ont été effectués. Les suites postopératoires étaient globalement satisfaisantes. Quoi que rare, le mélanome malin vulvaire est une affection à laquelle il faut penser en présence des facteurs de risque et toute masse suspecte. Il serait important d’en informer les patientes âgées sur les manifestations cliniques.
